# Meta-analysis of locking plate versus intramedullary nail for treatment of proximal humeral fractures

**DOI:** 10.1186/s13018-015-0242-4

**Published:** 2015-09-15

**Authors:** Guoqi Wang, Zhi Mao, Lihai Zhang, Licheng Zhang, Yanpeng Zhao, Peng Yin, Ling Gao, Peifu Tang, Hongjun Kang

**Affiliations:** Department of Orthopedics, Chinese PLA General Hospital, No. 28 Fuxing Road, Beijing, 100853 People’s Republic of China; Department of Critical Care Medicine, Chinese PLA General Hospital, No. 28 Fuxing Road, Beijing, 100853 People’s Republic of China; Medical College, Nankai University, No. 94 Weijin Road, Tianjin, 300071 People’s Republic of China; Department of Cardiovascular Surgery, Chinese PLA General Hospital, No. 28 Fuxing Road, Beijing, 100853 People’s Republic of China

**Keywords:** Proximal humeral fractures, Locking plate, Intramedullary nail, Meta-analysis

## Abstract

**Purpose:**

This meta-analysis compared the clinical outcomes of locking plate with intramedullary nail in the treatment of displaced proximal humeral fractures.

**Methods:**

We searched PubMed, Embase, and the Cochrane databases for studies comparing locking plate and intramedullary nail treatment of displaced two-, three-, or four-part proximal humeral fractures. The quality of the studies was assessed, and meta-analysis was performed using the Cochrane Collaboration’s REVMAN 5.1 software.

**Results:**

A total of 615 patients from eight studies were included in this meta-analysis (348 fractures treated with locking plate and 267 with intramedullary nail). Similar Constant scores were observed between the locking plate and intramedullary nail both in randomized controlled trials (RCTs) (mean difference (MD) = 2.12, 95 % confidence interval (CI), −2.54 to 6.79, *P* = 0.37) and observational studies (MD = −1.93, 95 % CI, −4.95 to 1.09, *P* = 0.21). Only one RCT provided American Shoulder and Elbow Surgeons Standardized scores indicating that the locking plate was better than the intramedullary nail (MD = 7.20, 95 % CI, 1.29–13.11, *P* = 0.02). The total complication rate did not specifically favor the locking plate or intramedullary nail both in the RCTs (risk ratio (RR), 2.44; 95 % CI, 0.35–16.78; *P* = 0.37) and observational studies (RR, 1.01; 95 % CI, 0.72–1.43; *P* = 0.94).

**Conclusions:**

In the existing literature, limited evidence suggests that the locking plate and intramedullary nail are both valuable options for the treatment of proximal humeral fractures. Because of the observed heterogeneity and variance between the subgroups, more RCT are needed to be able to definitively recommend a locking plate or intramedullary nail for specific fracture patterns.

## Introduction

Proximal humeral fractures are becoming increasingly prevalent with the rapidly expanding population, accounting for 6 % of all fractures in the human body [1]. Most proximal humeral fractures can be treated non-operatively, including a period of immobilization in an arm sling, followed by functional exercises [2]. However, to achieve satisfactory functional outcomes, displaced and unstable fractures are normally treated surgically [3]. It was reported that such fractures account for approximately 12.6 % of all proximal humeral fractures [4]. Many surgical strategies proved to be clinically effective, including locking plates, intramedullary nails, hemiarthroplasty, and reverse total shoulder replacement [5–7]. In 2013, Gomberawalla et al. [8] performed a meta-analysis comparing the joint preservation and arthroplasty for the treatment of displaced three- and four-part fractures of the proximal humerus. Patients in the joint-preserving groups displayed a significantly higher Constant score. Dai et al. [9] compared locking plate fixation with hemiarthroplasty for complex proximal humeral fractures and concluded that patients with a locking plate fixation could achieve more favorable functional outcomes. Handoll [10] reviewed two studies that compared the locking plate and intramedullary nail. However, no systematic review or meta-analysis was available for the outcomes between the locking plate and intramedullary nail for proximal humeral fractures. Several studies [6, 11–17] have focused on locking plate and intramedullary nail treatment of displaced two-, three-, and four-part proximal humeral fractures. However, an integral body of evidence was urgent regarding the outcomes of locking plate and intramedullary nail treatment for proximal humeral fractures because of advances in the methods of internal fixation devices.

In the present study, we performed a meta-analysis on the available evidence to evaluate the effects of locking plate and intramedullary nail treatments for proximal humeral fractures on the clinical outcomes and complications. We hypothesized that the locking plate and intramedullary nail would display similar results regarding functional outcomes and complications.

## Material and methods

### Search strategy

A search of the Pubmed (1966–October 2014), Embase (1980–October 2014), and Cochrane databases (1966–October 2014) was performed for eligible trials. We combined the search terms “proximal humeral/humeral fracture”, “internal fixation/locking plate fixation”, and “intramedullary nail/nail”. Additional strategies to screen relevant literature were supplemented using Google Scholar or scan reference lists from identified trials and review articles. No language restriction was made.

### Inclusion and exclusion criteria

We included studies when the following criteria were met: (1) Comparative studies of level III and higher, including displaced proximal humerus fractures allocated into two treatment groups: (a) IMN group and (b) locking plate group; (2) the outcome measures included functional scores, method-related complications, and additional surgery data; and (3) studies in which a follow-up of a minimum of 6 months was involved. Excluded studies were the following: (1) abstracts, letters, and meeting proceedings; (2) repeated data; and (3) patients with pathologically or metabolically induced fractures or open fractures.

### Data extraction and quality assessment

Two authors independently identified the appropriate articles. Data including patient characteristics (mean age, female rate), study type, interventions, time to last follow-up, follow-up rate, inclusion criteria, function score, and complications would be extracted from the included articles. Disagreements were discussed and when not resolved, a third author was consulted.

### Assessment of methodological quality

We evaluated the randomized controlled trials (RCTs) using the “Cochrane collaboration’s tool for assessing the risk of bias,” which included the following aspects: (1) random-sequence generation (selection bias), (2) allocation concealment (selection bias), (3) blinding of participants and personnel (performance bias), (4) blinding of outcome assessment (detection bias), (5) incomplete outcome data (attrition bias), (6) selective reporting (reporting bias), and (7) other bias. The methodological qualities of the non-RCTs were assessed independently by two authors using the methodological index for non-randomized studies (MINORS) [18]. The MINORS is a valid instrument used to assess the methodological qualities of non-randomized surgical studies, including observational studies. In this meta-analysis, a MINORS score >12 was considered the level for inclusion.

### Statistical analysis

The data from the studies were entered into the Cochrane Collaboration’s REVMAN 5.1 software. A *P* value <0.05 was considered statistically significant. A visual forest plot was used to evaluate heterogeneity, and the test for heterogeneity and the *I*^2^ statistic [19] was considered at the same time. An *I*^2^ value >50 % was considered to indicate substantial heterogeneity. A fixed-effects model was used in the meta-analysis. However, a random-effects model was used when significant heterogeneity among the studies was found. The random-effects model of DerSimonian and Laird [20] was used regardless of heterogeneity. Continuous variables were presented as the mean difference (MD), and dichotomous variables were presented as the risk ratio (RR), both with 95 % confidence intervals (CIs).

Sensitivity analysis would be conducted by omission of each single study to evaluate stability of the results if heterogeneous studies existed.

## Results

### Study selection and characteristics

**Fig. 1:**
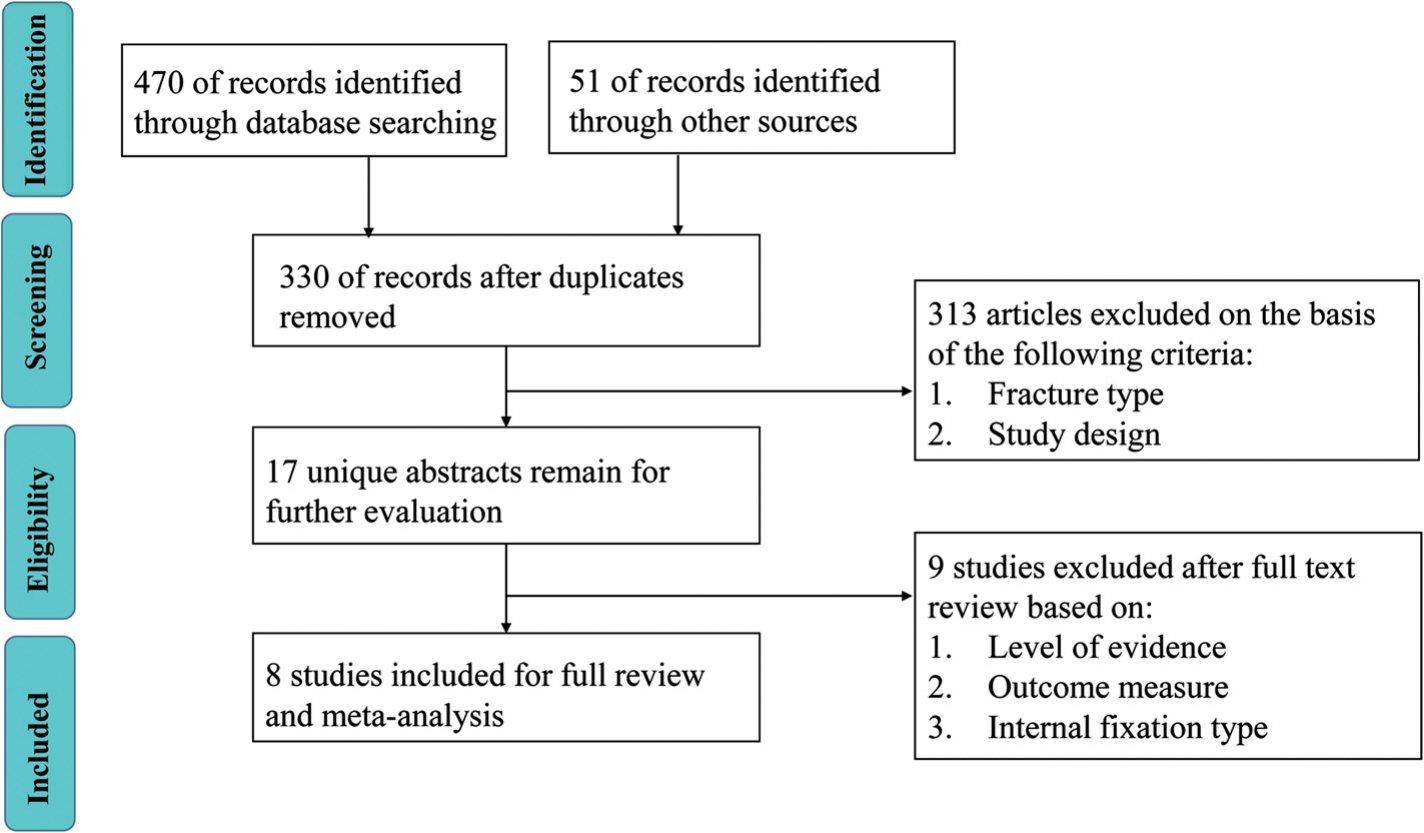
Flow diagram shows the process of literature selection

We initially identified 521 relevant articles. Eight papers met our inclusion criteria. The flowchart of Figure 1 depicts the results of the search process and the finally recruited eligible studies. Two RCTs [6, 13], two prospective comparative studies [11, 16], and four retrospective comparative studies [12, 14, 15, 17] were included (Fig. 1). These studies included a total of 615 patients, of whom 348 were in the locking plate group and 267 in the intramedullary nail group. The total number of patients in each study varied from 22 to 211, while the mean ages had a range of 50.5–68.3 years. The percentage of female patients in the study populations varied from 36.1 to 83.3 %. The patient follow-up periods were over 12 to 36 months, while the follow-up rate varied from 74.8 to 100 %. Table 1 summarizes the characteristics of each of the studies included.

**Table 1:** Characteristics of included studies

Studies	Intervention		Mean age (year) (LP *V*. IN)	Number (LP *V*. IN)	Percent female (%)	Follow-up (month)	Rate of follow-up	Type of study	Diagnosis characteristics
	LP	IN							
Von et al. 2014 [17]	Locking plate osteosynthesis (PHILOS®, Synthes, Umkirch, Deutschland; VariAX®, Stryker, Duisburg, Deutschland)	Intramedullary nailing (T2-PHN®, Stryker, Duisburg, Deutschland)	61 (35–84)	28 *V*. 44	36.1	38–82	100	Retrospective	Displaced three- or four-part fractures
Lekic et al. 2011 [15]	Locking plates osteosynthesis (Stryker, Mahwah, NJ; Accumed, Trenton, NJ)	Intramedullary nailing (Synthes, Paoli, PA)	59 (21–81) *V*. 60 (37–83)	12 *V*. 11	83.3 *V*. 54.5	3–46	92	Retrospective	Displaced two-part fractures
Konrad et al. 2012 [16]	Plate (proximal humeral interlocking system (PHILOS)/locking proximal humerus plate (LPHP))	Nail (proximal humeral nail (PHN))	65.4 (15.6) *V*. 64.8 (13)	153 *V*. 58	*81 V*. 74	3–12	84.4	Prospective	Displaced three-part fractures
Trepat et al. 2011 [14]	PHILOS plate	NHP-T2 nail	68.3 (17.3) *V*. 64.5 (20.7)	14 *V*. 15	72.7 *V*. 53.8	6–12	82.8	Retrospective	Displaced two-part fractures
Zhu et al. 2011 [6]	Locking plate osteosynthesis (LPHP; Synthes; PHILOS; Synthes)	Locking intramedullary nail (PHN; Synthes, Oberdorf, Switzerland)	50.5 (19.9) *V*. 54.8 (17.1)	26 *V*. 25	69.2 *V*. 64.0	12–36	89.0	RCT	Displaced two-part fractures
Smejkal et al. 2011 [13]	PHILOS plate (Synthes, Switzerland)	Intramedullary nails (Zifko method)	61 (21–81)	28 *V*. 27	81.8	2–18	90.2	RCT	Displaced two or three-part fractures
Matziolis et al. 2010 [12]	Locking compression plate (PHP)	Zifko nails	54.8 (22–72) *V*. 55.6 (16–74)	11 *V*. 11	63.6 *V*. 63.6	36	100	Retrospective	Displaced two-part fractures
Gradl et al. 2009 [11]	Locking Proximal Humerus Plate (Mathys AG, Bettlach, Switzerland)	Sliding stable interlocking nail (Targon PH; B. Braun-Aesculap, Tuttlingen, Germany)	63 (16)	76 *V*. 76	68.4	12	74.8	Prospective	Displaced two-, three-, or four-part fractures

*LP* locking plate, *IN* intramedullary nail

### Methodological quality

**Fig. 2:**
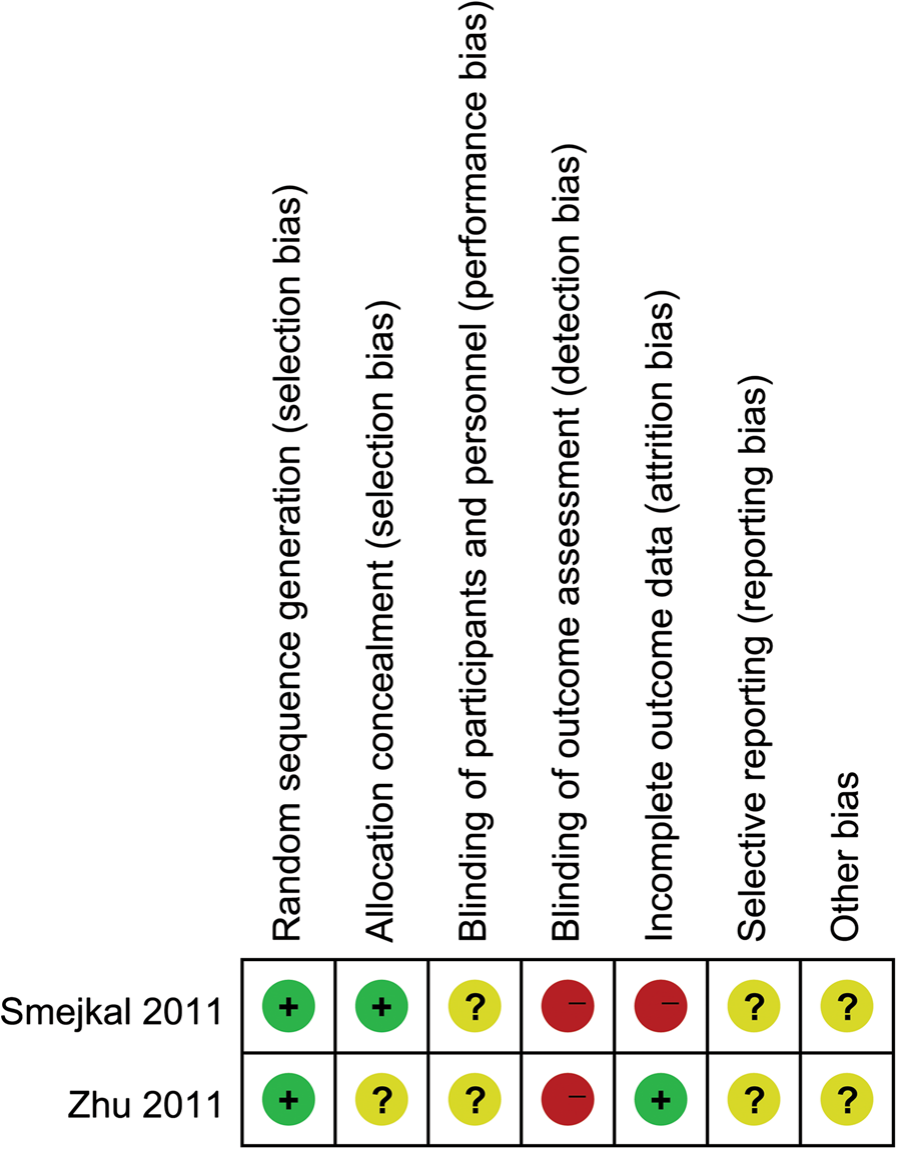
The methodological quality of the RCTs. Risk of bias summary. “+” means low risk; “?” means unclear risk; “-” means high risk

The methodological quality of the RCTs is presented in Fig. 2. Six non-randomized trials were assessed using the MINORS score (Table 2). One study scored 14, two scored 16, one scored 17, and two scored 18. Although a risk of bias was found in all the studies, this was moderate throughout.

**Table 2:** MINORS appraisal scores for the included retrospective studies

Study	Methodologic items^a^												Total
	1	2	3	4	5	6	7	8	9	10	11	12	
Von et al. 2014	2	1	0	2	0	2	2	0	1	2	2	2	16
Lekic et al. 2012	2	2	0	2	0	2	1	0	1	0	2	2	14
Konrad et al. 2012	2	2	2	2	0	2	1	0	1	2	2	2	18
Trepat et al. 2011	2	2	0	2	0	2	1	0	1	2	2	2	16
Matziolis et al. 2010	2	2	0	2	0	2	2	0	1	2	2	2	17
Gradl et al. 2009	2	2	2	2	0	2	1	0	1	2	2	2	18

^a^Methodologic items are as follows: (1) a clearly stated aim; (2) inclusion of consecutive patients; (3) prospective collection of data; (4) endpoints appropriate to the aim of the study; (5) unbiased assessment of the study endpoint; (6) follow-up period appropriate to the aim of the study; (7) loss to follow-up, which is less than 5 %; (8) prospective calculation of the study size; (9) an adequate control group; (10) contemporary groups; (11) baseline equivalence of groups; and (12) adequate statistical analyses. The items are scored as “0” (not reported), “1” (reported but inadequate) or “2” (reported and adequate). The global ideal score for comparative studies is 24 [18]

### Effects of locking plate vs. intramedullary nail

**Fig. 3:**
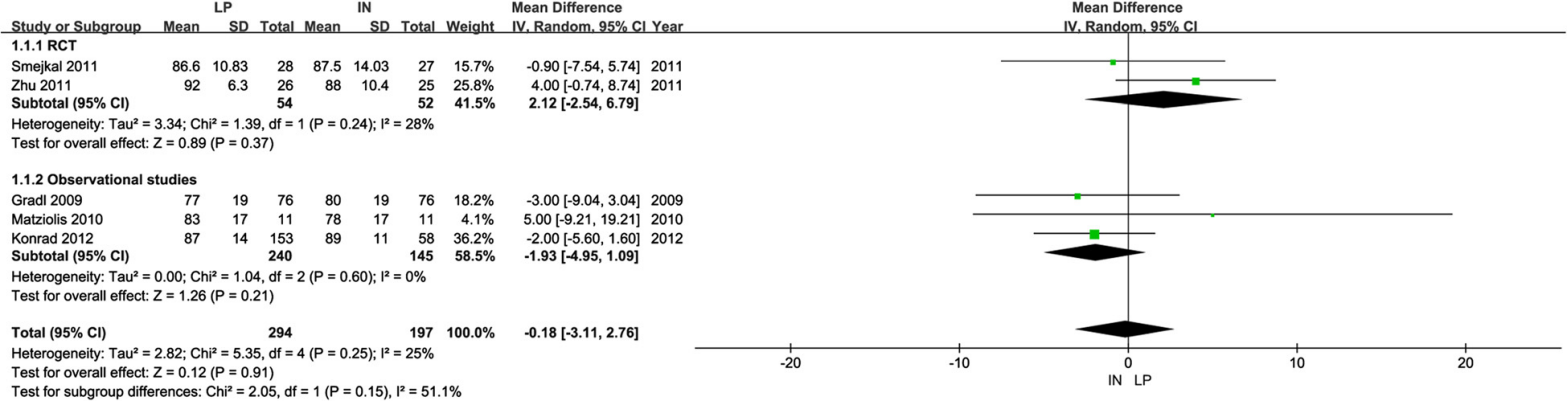
Meta-analysis of Constant scores: subgroup analyses. *LP* locking plate, *IN* intramedullary nail

The Constant score was not significantly different between the locking plate and intramedullary nail groups using a random-effect model both in the RCTs (MD = 2.12, 95 % CI, −2.54 to 6.79, *P* = 0.37, Fig. 3) and observational studies (MD = −1.93, 95 % CI, −4.95 to 1.09, *P* = 0.21, Fig. 3). The locking plate displayed a better American Shoulder and Elbow Surgeons Standardized (ASES) score compared with the intramedullary nail for displaced two-part fractures. (MD = 7.20, 95 % CI, 1.29–13.11, *P* = 0.02).

**Fig. 4:**
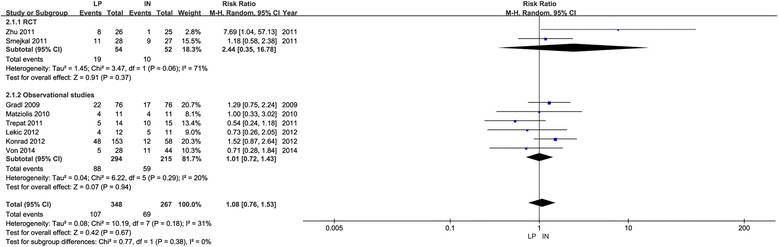
Forest plot for total complication rate between locking plate group and intramedullary nail group. *LP* locking plate, *IN* intramedullary nail

All eight studies included in this meta-analysis provided information on the total complication events or presented them in a tabular form. Meta-analysis of the total complication events displayed no significant difference between the locking plate and intramedullary nail (RR, 1.08; 95 % CI, 0.76–1.53; *P* = 0.67, heterogeneity test, *P* = 0.38, *I*^2^ = 0 %, Fig. 4). Subgroup analysis of the RCTs (RR, 2.44; 95 % CI, 0.35–16.78; *P* = 0.37, Fig. 4) and observational studies (RR, 1.01; 95 % CI, 0.72–1.43; *P* = 0.94, Fig. 4) also failed to find a significant difference between the locking plate and intramedullary nail. There were no statistically significant differences in the rates of additional surgery, osteonecrosis, infection, nonunion, penetration, impingement, and fracture redisplacement between the locking plate and intramedullary nail groups (Table 3).

**Table 3:** Complications reported

Outcomes	No. of trials	No. of patients	Plate group	Nail group	RR (95 % CI)	*P* value for RR	*I* ^2^, %	*P* value for heterogeneity
Additional surgery	8 [6, 11–17]	615	48 of 348	37 of 267	0.98 (0.65, 1.47)	0.92	11	0.34
Osteonecrosis	4 [11, 12, 15, 16]	408	6 of 252	5 of 156	0.93 (0.32, 2.75)	0.90	0	0.66
Infection	4 [12, 13, 16, 17]	360	6 of 220	1 of 140	2.09 (0.49, 8.90)	0.32	0	0.81
Nonunion	3 [11, 14, 16]	295	7 of 195	1 of 100	2.24 (0.50,10.14)	0.29	0	0.41
Penetration	6 [6, 11, 13–16]	521	25 of 309	10 of 212	1.59 (0.79, 3.18)	0.19	25	0.25
Impingement	3 [13, 14, 16]	295	8 of 195	6 of 100	0.89 (0.11, 7.02)	0.91	55	0.11
Redisplacement of fracture	2 [11, 17]	224	5 of 104	8 of 120	0.80 (0.02, 39.17)	0.91	79	0.03

*CI* confidence interval, *RR* risk ratio

### Sensitivity analysis

Sensitivity analysis showed that omitting any single study did not significantly affect the pooled RR or MD (Tables 4 and 5).

**Table 4:** Sensitivity analyses based on various exclusion criteria for total complication

Excluded trial	No. of trials	No. of patients	Plate group	Nail group	RR (95 % CI)	*P* value for RR	*I* ^2^, %	*P* value for heterogeneity
Zhu 2011	7 [11–17]	564	99 of 322	68 of 242	1.06 (0.80, 1.41)	0.68	5	0.39
Smejkal 2011	7 [6, 11, 12, 14–17]	560	96 of 320	60 of 240	1.05 (0.69, 1.60)	0.80	41	0.12
Gradl 2009	7 [2, 12–17]	463	85 of 272	52 of 191	1.03 (0.67, 1.58)	0.91	39	0.13
Matziolis 2010	7 [6, 11, 13–17]	593	103 of 337	65 of 256	1.08 (0.73,1.60)	0.70	41	0.12
Trepat 2011	7 [6, 11–13, 15–17]	586	102 of 334	59 of 252	1.22 (0.90, 1.65)	0.20	5	0.39
Lekic 2012	7 [6, 11–14, 16, 17]	592	103 of 336	64 of 256	1.12 (0.76, 1.64)	0.56	37	0.15
Konrad 2012	7 [6, 11–15, 17]	404	59 of 195	57 of 209	0.99 (0.66, 1.47)	0.95	30	0.20
Von 2014	7 [6, 11–16]	543	102 of 320	58 of 223	1.13 (0.77, 1.65)	0.53	36	0.15

*CI* confidence interval, *RR* risk ratio

**Table 5:** Sensitivity analyses based on various exclusion criteria for total complication

Excluded trial	No. of trials	MD (95 % CI)	*P* value for MD	*I* ^2^, %	*P* value for heterogeneity
Smejkal 2011	4 [6, 11, 12, 16]	0.06 (−3.72, 3.84)	0.97	44	0.15
Zhu 2011	4 [11–13, 16]	−1.76 (−4.51, 0.99)	0.21	0	0.77
Gradl 2009	4 [6, 12, 13, 16]	0.52 (−2.96, 4.00)	0.77	33	0.22
Matziolis 2010	4 [6, 11, 13, 16]	−0.39 (−3.58, 2.80)	0.81	37	0.19
Konrad 2012	4 [6, 11–13]	0.85 (−2.90, 4.60)	0.66	22	0.28

*CI* confidence interval, *MD* mean difference

### Publication bias

**Fig. 5:**
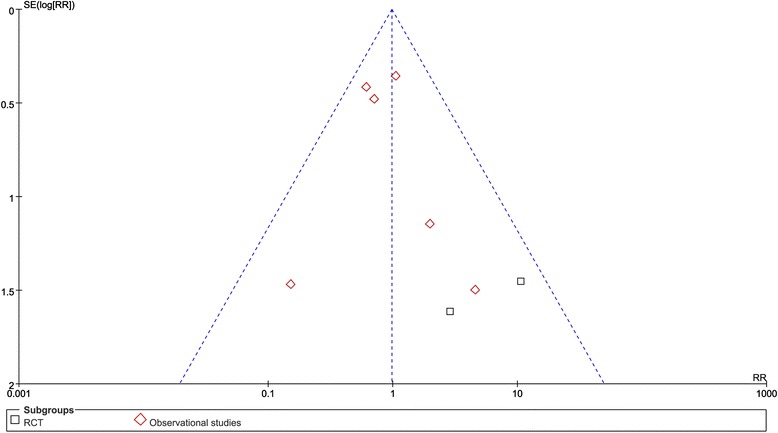
Funnel plot for publication bias

For the meta-analysis of additional surgery, there was no evidence of significant publication bias by inspection of the funnel plot (Fig. 5).

## Discussion

To our knowledge, this is the first systematic review and meta-analysis comparing the locking plate and intramedullary nail in the treatment of two-, three-, and four-part proximal humeral fractures. We reviewed eight articles comparing the clinical results of the locking plate and intramedullary nail, restricting our study to level III or higher studies. According to the inclusion and exclusion criteria of the study design, although the participants included were not restricted to older patients, the mean age of the patients was >50 years. Our results displayed similar effects of the locking plate and intramedullary nail on the Constant score and the rates of total complication, additional surgery, osteonecrosis, and other complications. With respect to the ASES score, only one RCT from 2011 presented data that favored the locking plate over the intramedullary nail regarding displaced two-part fractures.

Operative intervention has proved an effective and safe treatment option for fractures of the proximal humerus [21, 22]. There are numerous optional fixation devices when surgeons attempt a proximal humeral fracture operation, including the locking plate, intramedullary nail, and artificial joint (hemiarthroplasty, total shoulder arthroplasty, and reverse shoulder arthroplasty) [23, 24]. In the majority of cases, arthroplasty options are considered for older adults because osteoporotic bone limits the ability to achieve stable internal fixation [25]. However, because of the possibility of a limited function, which could influence the quality of life, controversy still surrounds the use of artificial joints [9].

Over the past decade, internal fixation has been the first option for displaced proximal humeral fractures. The Constant score is always used for shoulder outcome evaluation after internal fixation [26]. Sproul [27] performed a meta-analysis (12 studies) of locking plate fixation of proximal humeral fractures, and the mean Constant score for the entire review population (514 patients) was 74, with high rates of complications and reoperation. However, only the English literature was evaluated in this review, and no comparison was made with other methods. A systematic literature review (12 studies) on the benefits and harm of locking plate osteosynthesis in intraarticular (Orthopaedic Trauma Association Type C) fractures of the proximal humerus was made in 2012, and in all the included studies, the mean non-adjusted Constant score varied from 53 to 75 [28]. However, these studies lacked randomized and comparative evaluation. In our systematic review, analyses of the Constant score failed to find any statistically significant differences for proximal humeral fractures between the locking plate and intramedullary nail. The treatment result of the ASES score indicated a significant difference favoring the locking plate for displaced two-part fractures. However, with only one study with this functional outcome, improved results require more RCTs.

There are considerable complications in locking plate fixation of the proximal humerus [29]. Brorson et al. [30] reported a complication rate of 16–64 % for locking plate treatment. Roderer found that implant-related complications occurred in 9 of 54 patients (17 %) with unstable proximal humeral fractures using the locking plate [31]. Osteoporotic bone and increasing age may increase the failure rate of the locking plate for proximal humeral fractures [32]. Kloub reported long-term results of the nailing of extra-articular proximal humeral fractures, with a low complication rate, and found that age had no influence on the final functional result [33]. A systematic review by Gupta et al. [34] evaluated the outcomes of four methods for complex proximal humeral fractures, including open reduction and internal fixation (ORIF) and closed reduction and percutaneous pinning (CRPP). There was a greater complication rate following CRPP compared with ORIF. However, this conclusion came from non-comparative studies and CRPP differed from the intramedullary nail. In our study, the total complication rates of the locking plate and intramedullary nail groups were 30.7 and 25.8 %, respectively, which were not statistically significantly different (RR, 1.08; 95 % CI, 0.76–1.53; *P* = 0.67). One of the major complications in the two groups was osteonecrosis, which was strongly correlated with a high risk of an initial dislocation and resulted in painful dysfunction of the shoulder. A similar low rate of osteonecrosis was found for the two methods (results not shown). Thanasas [35] reported a reoperation rate of 13.7 % for the locking plate in the treatment of proximal humeral fractures. Any complication that required additional surgery had been recorded in this meta-analysis, and very similar results were found for the locking plate (13.7 %) and intramedullary nail (13.8 %). The additional surgery rate for the locking plate in our study was the same as in Thanasas [35]. Other complications, including infection, nonunion, and screw cutout, failed to display any statistically significant differences.

Our study had a number of limitations. First, only eight articles were included, of which only two were RCTs with a total of 106 fractures that could provide level I evidence. Second, it was difficult to obtain sufficient statistical power to make any conclusions regarding clinically important differences, because a moderate risk of bias was observed on the basis of the MINORS score. Third, the follow-up of most articles in the present study was <2 years. Therefore, the data from these articles were inadequate for interpreting long-term results. Considering the limitations of the reviewed studies, more large well-designed RCTs that incorporate the long-term evaluation of clinically relevant outcomes in participants with different underlying risks of shoulder function are required to better assess the roles of the locking plate and intramedullary nail.

## Conclusion

Current limited evidence indicates that the locking plate and intramedullary nail are both valuable options for the treatment of displaced two-, three-, and four-part proximal humeral fractures in older patients. They displayed similar Constant scores and complication rates. The main advantage of the locking plate was the higher ASES score for displaced two-part proximal humeral fractures. Because of the modest sample size and only a short-term follow-up, our findings should be interpreted cautiously.
